# Comparative Proteomic Analysis of Light-Induced Mycelial Brown Film Formation in* Lentinula edodes*

**DOI:** 10.1155/2016/5837293

**Published:** 2016-10-27

**Authors:** Li Hua Tang, Qi Tan, Da Peng Bao, Xue Hong Zhang, Hua Hua Jian, Yan Li, Rui heng Yang, Ying Wang

**Affiliations:** ^1^National Engineering Research Centre of Edible Fungi, Key Laboratory of Edible Fungi Resources and Utilization (South), Ministry of Agriculture, Institute of Edible Fungi, Shanghai Academy of Agricultural Sciences, Shanghai 201403, China; ^2^State Key Laboratory of Microbial Metabolism, School of Life Sciences and Biotechnology, Shanghai Jiao Tong University, Shanghai 200240, China

## Abstract

Light-induced brown film (BF) formation by the vegetative mycelium of* Lentinula edodes* is important for ensuring the quantity and quality of this edible mushroom. Nevertheless, the molecular mechanism underlying this phenotype is still unclear. In this study, a comparative proteomic analysis of mycelial BF formation in* L. edodes* was performed. Seventy-three protein spots with at least a twofold difference in abundance on two-dimensional electrophoresis (2DE) maps were observed, and 52 of them were successfully identified by matrix-assisted laser desorption/ionization tandem time-of-flight mass spectrometry (MALDI-TOF/TOF/MS). These proteins were classified into the following functional categories: small molecule metabolic processes (39%), response to oxidative stress (5%), and organic substance catabolic processes (5%), followed by oxidation-reduction processes (3%), single-organism catabolic processes (3%), positive regulation of protein complex assembly (3%), and protein metabolic processes (3%). Interestingly, four of the proteins that were upregulated in response to light exposure were nucleoside diphosphate kinases. To our knowledge, this is the first proteomic analysis of the mechanism of BF formation in* L. edodes*. Our data will provide a foundation for future detailed investigations of the proteins linked to BF formation.

## 1. Introduction

The edible medicinal mushroom species* Lentinula edodes* ranks second in production among all mushrooms in the world and is an important source of not only food but also lentinan that has medicinal value. During cultivation of this mushroom, the formation of brown film (BF) by the mycelia is an important stage before primordia and fruiting formation, and it is closely related to mushroom quality. BF formation is affected by many environmental factors, including light. However, the mechanism of light-induced brown film formation is poorly understood, and the associated changes in protein expression have not been explored.

The rapidly developing field of proteomics has become increasingly relevant to the study of fungal biology. Proteomics is a powerful tool used for the sensitive detection and rapid identification of changes in protein expression in response to various biotic and abiotic stresses [1–3] and has been utilized in many studies on the physiology and development of filamentous fungi [4]. In addition, proteomics has been applied to study the growth and development of various types of mushrooms such as* L. edodes* [5]. Other proteomic studies have evaluated the effects of low-temperature stress on protein expression in the mycelium and fruiting body of* Flammulina velutipes* [6], the effects of light on the growth and development of the cap and stipe of* F. velutipes* [7], differential protein expression in dual-core and single-core mycelial hyphae and in the fruit body of* Flammulina velutipes* [8], and protein expression during the fruiting of* Hericium erinaceus* and* Sparassis crispa* [9]. However, proteomics has not been applied to investigate light-induced BF formation in the* L. edodes* mycelium.

Although the biology and physiology of light-induced BF formation have been studied previously [10], little is known about the changes in protein expression associated with this process. In this study, we used two-dimensional electrophoresis (2-DE) and matrix-assisted laser desorption/ionization tandem time-of-flight mass spectrometry (MALDI-TOF/TOF/MS) to identify proteins differentially expressed in mycelia with and without BF formation. The results of this study provide insights into the molecular mechanisms and physiological basis of light-induced BF formation in* L. edodes*, as well as mushroom development and secondary metabolite production, which will facilitate the development of methods for promoting BF formation and improving the quality of the fruit body of mushrooms.

## 2. Materials and Methods

### 2.1. Cultivation of Fungal Mycelia

*L. edodes* strain 135 was obtained from the Agricultural Culture Collection of China (Beijing) (number ACCC50903). Fungal mycelia were grown at 22°C in cultivation bags containing 850 g of medium (80% dry sawdust and 20% dry corn bran) in the dark for 30 d, after which the substrate was fully colonized. Bags were then either exposed to a 12-h light/dark protocol (white light, 300 lx) for 50 d to induce BF formation on the mycelial surface (sample 313C) or maintained for 50 d in the dark (sample 313W, controls). Samples from the mycelial surface were then collected for protein preparation.

### 2.2. Protein Extraction

Protein extraction was performed using the trichloroacetic acid- (TCA-) acetone method as previously described [11], with some modifications. The samples were ground to a powder in liquid nitrogen, and 1 g of sample powder was extracted in 10 mL of 10% TCA in cold acetone at −20°C for 1 h. After centrifugation at 15,000 ×g for 15 min at 4°C, the deposits were washed three times with 10 mL of cold acetone at −20°C for 1 h and centrifuged at 15,000 ×g for 15 min at 4°C. The deposits were then collected and dried in a vacuum freeze dryer. The deposits were dissolved in a lysis solution containing 9 M urea, 4% CHAPS (w/v), 1% DTT (w/v), and 1% IPG buffer (v/v) (pH 3–10, GE Healthcare Bio-Science, Little Chalfont, UK) at 30°C for 1 h, and the solution was centrifuged at 15,000 ×g for 15 min at room temperature. The supernatants were collected and centrifuged again, and the concentration of the protein extracts was determined by the Bradford method [12]. The extracted protein solution was stored at −80°C until isoelectric focusing.

### 2.3. 2DE Image and Data Analysis

Samples containing 300 *μ*g of proteins were brought to a total volume of 450 *μ*L in fresh rehydration buffer (9 M urea, 4% CHAPS, 1% DTT, 1% IPG buffer, and a trace amount of bromophenol blue) and loaded on a DryStrip gel (GE Healthcare, 24 cm, pH = 3–10, NL). Isoelectric focusing was performed with an IPGphor system (GE Healthcare). The voltage was set to 50 V for 12 h, 500 V for 1 h, 1,000 V for 1 h, 10,000 V for 1 h, and 10,000 V for 8 h. The strips were subsequently incubated in equilibration buffer (6 M urea, 30% glycerol, 2% SDS, 50 mM Tris-HCl (pH 8.8), 1% DTT, and a trace amount of bromophenol blue) for 15 min followed by 15 min in 2.5% (w/v) iodoacetamide in equilibration buffer. Then, the IPG strips were loaded in a 12% (w/v) SDS-PAGE gel using the Ettan Dalt Six system (GE Healthcare). The gels were run at 100 V for 1 h and maintained at 200 V until the dye front reached the bottom of the gel. The gel was visualized by silver staining according to Yan's protocol [13] and scanned by Image Scanner software (GE Healthcare, USA) at a resolution of 300 dots per inch. The gel images were processed in three steps using PDquest 8.0 software (Bio-Rad): spot detection, volumetric quantification, and matching. A total of six 2DE gels resulting from three independent biological replicates of both the 313W and 313C sample were analyzed and the standard deviations were calculated from the three independent replicates. A threshold of *p* ≤ 0.05 and a fold change of ≥2 or ≤0.5 were used to identify significant differentially expressed protein spots.

### 2.4. Protein Digestion

Gel spots were excised and destained with 15 mM K_3_Fe(CN)_6_ in 50 mM NaS_2_O_3_ at room temperature for 5 min. After removal of the destaining solution, the gels were washed twice with 200 *μ*L of ddH_2_O, and 50 *μ*L of 25 mM NH_4_HCO_3_ and 100 *μ*L of 50% ACN were then added, followed by 100 *μ*L of 100% ACN. The gels were rehydrated in 5 *μ*L of trypsin solution (Promega, Madison, WI, USA; 20 *μ*g/mL in 25 mM NH_4_HCO_3_) for 30 min. Next, 20 *μ*L of cover solution (25 mM NH_4_HCO_3_) was added, and the gels were digested for 16 h at 37°C. The supernatants were transferred to another tube, and the gels were extracted once with 50 *μ*L of extraction buffer (67% ACN and 5% TFA). The peptide extracts and gel spot supernatants were combined and then completely dried.

### 2.5. Protein Identification by MALDI-TOF/TOF/MS and Database Search

Samples were resuspended in 5 *μ*L of 0.1% TFA and mixed with a matrix comprising a saturated solution of *α*-cyano-4-hydroxy-trans-cinnamic acid (CHCA) in 50% ACN/0.1% TFA (1 : 1). Then, 1 *μ*L of the mixture was spotted on a stainless steel sample target plate. Peptide MS and MS/MS were performed with an ABI 5800 MALDI-TOF/TOF Plus mass spectrometer (Applied Biosystems, Foster City, CA, USA). Data were acquired with a positive MS reflector using a CalMix5 standard to calibrate the instrument (ABI5800 Calibration Mixture). Both the MS and MS/MS data were integrated and processed by GPS Explorer V3.6 software (Applied Biosystems, USA) with default parameters. Based on the combined MS and MS/MS spectra, proteins were successfully identified based on the 95% or higher confidence interval of their scores in the MASCOT V2.3 search engine (Matrix Science Ltd., London, UK) with the following search parameters: a local database with peptide sequences created from the mycelial transcriptome (RNA-Seq) of* L. edodes* [10], trypsin as the digestion enzyme, one missed cleavage site, partial modifications of cysteine carbamidomethylation and methionine oxidization, no fixed modifications, and 200 ppm for precursor ion tolerance and 0.4 Da for fragment ion tolerance.

### 2.6. Quantitative Real-Time PCR (RT-qPCR) Validation of Proteomic Data

Eleven proteins exhibiting significant upregulation in proteomic analysis (313C/313W) were selected for validation using RT-qPCR. cDNAs were synthesized according to the manufacturer's protocol (Takara, Dalian, China) and used as templates for real-time PCR using specific primers (Table  S1 in Supplementary Material available online at http://dx.doi.org/10.1155/2016/5837293). Real-time PCR was performed using the FTC2000 System (Canada). Reaction mixtures (50 *μ*L) contained 0.5 *μ*L 20x SYBR green Mastermix, 1 *μ*L of each primer (25 pmol/*μ*L), 2 *μ*L 10-fold diluted cDNA template, and 20.5 *μ*L DEPC water. Amplification conditions were as follows: 94°C for 4 min, 35 cycles of 94°C for 20 s, 60°C for 30 s, and 72°C for 30 s. Each reaction was performed in triplicate using 18S rRNA as the internal control gene, and the relative gene expression levels were determined using the delta-delta Ct method [14].

## 3. Results

### 3.1. Growth of the* L. edodes* Mycelium

**Figure 1:**
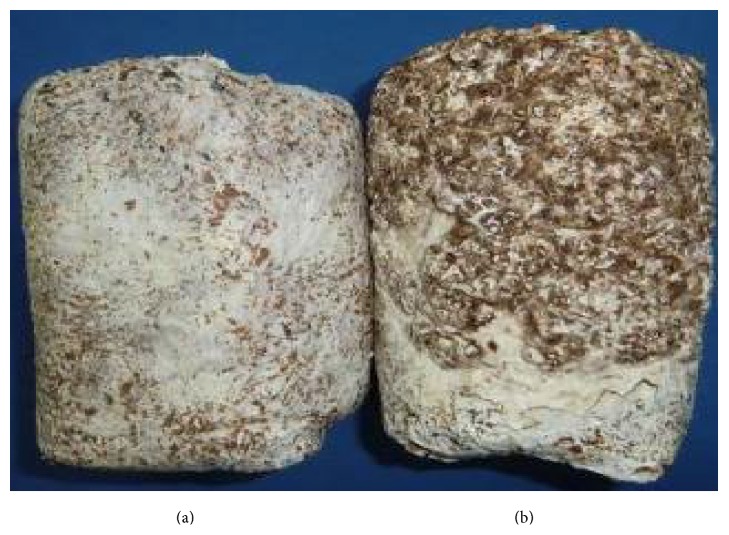
Comparison of the surface of the mycelium grown under different illumination conditions. (a) 50 d of 24 h of darkness; (b) 50 d of a 12 h dark/light regimen (sample 313C).

The cultivation substrate was fully colonized by white fungal mycelia after incubation of the test bags for 30 d in the dark. In the test bags that were subsequently exposed to a 12 h light/dark protocol, the mycelia gradually developed a BF (sample 313C; Figure 1(b)), whereas the mycelia in the control group remained white (sample 313W; Figure 1(a)).

### 3.2. 2DE Analysis of the Mycelial Protein Profile

**Figure 2:**
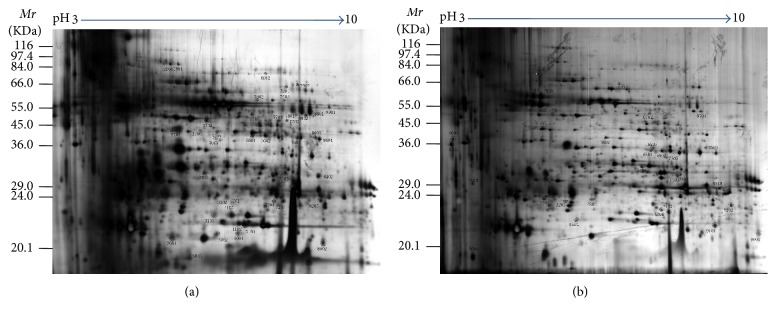
Representative 2DE protein patterns from* Lentinula edodes *mycelium sample 313W that did not produce a brown film (a) and sample 313C (b) that produced a brown film. 313W indicates samples that did not form a brown film when cultivated in the dark, while 313C denotes samples that formed a brown film when cultivated under light/dark conditions. Proteins were loaded on a 24 cm IPG strip with a nonlinear immobilized pH gradient ranging from 3 to 10 for isoelectric focusing, followed by electrophoresis in a 12% SDS-PAGE gel and silver staining.

To identify proteins that were differentially expressed during mycelial BF formation in response to light, a comparative proteomic analysis was performed on the mycelial surface components of the brown film and control groups. The extracted proteins were resolved and analyzed by 2DE with a pH range of 3 to 10, and the gels were visualized by silver staining and analyzed with PDquest software. Two representative images of the 2DE gel are shown in Figure 2; according to this figure, there were 789 ± 14 and 726 ± 30 examined protein spots for 313W and 313C, respectively, which demonstrates that more than 650 protein spots were detected, and most of the protein spots were distributed in the Mr range of 20.1 to 84.0 kDa. Of these spots, a total of 73 stained spots had significant and repeatable changes in abundance when the two treatments were compared (313C versus 313W).

### 3.3. Identification of Differentially Expressed Proteins (DEPs)

Among these 71 protein spots, 52 were successfully identified by MALDI-TOF/TOF, and a local protein database was constructed from the recently sequenced mycelium transcriptome [10]. Among the 52 proteins, 23 were upregulated and 29 were downregulated when 313C was compared to 313W. Moreover, 23 proteins were upregulated (Table  S2); for example, small heat shock proteins such as heat shock cognate 70, spot 7404, which was induced by light treatment during BF formation, was upregulated compared to the control. Meanwhile, two proteins (20S proteasome subunit, spot 6504, and proteasome component pts1, spot7505) involved in proteasome were also upregulated in 313C. Short-chain dehydrogenase/reductase (SDR, spot 0301) was 3.61-fold upregulated in 313C compared to 313W, and manganese superoxide dismutase (spot 2202) was 2.39-fold upregulated in 313C compared to 313W. Four nucleoside diphosphate kinase proteins (spot 0006, spot 7102, spot 6204, and spot 9202) were all ≥2-fold upregulated in 313C compared to 313W, while for 29 proteins their expression was significantly downregulated in 313C, including NAD-aldehyde dehydrogenase (spot 6602), protein disulfide isomerase (spot 8402), succinate-semialdehyde dehydrogenase (spot 7702), heat shock protein (spot 5704), glycoside hydrolase family 13 protein (spot 5902), proliferation-associated 2G4 (spot 9901), tripeptidyl peptidase A (spot 8801), heat shock cognate 70 (spot 3704), hsp70-like protein (spot 6902), small ubiquitin-related modifier (spot 7203), and FG-GAP repeat-containing protein (spot 2904). Carbohydrate-binding module family 13 protein (spot 8002) was more than 9-fold downregulated in 313C. Some proteins were distributed across multiple spots at different positions on the same gel, including adenosine kinase (spots 2909 and 2910), heat shock cognate 70 (spots 3704, 3705, and 4201), and nucleoside diphosphate kinase (spots 0006, 7102, 6204, and 9202). The multiple spots suggest that these proteins may be subjected to posttranslational modifications, such as phosphorylation, N-acetylation, and glycosylation.

### 3.4. Functional Classification of the DEPs

**Figure 3:**
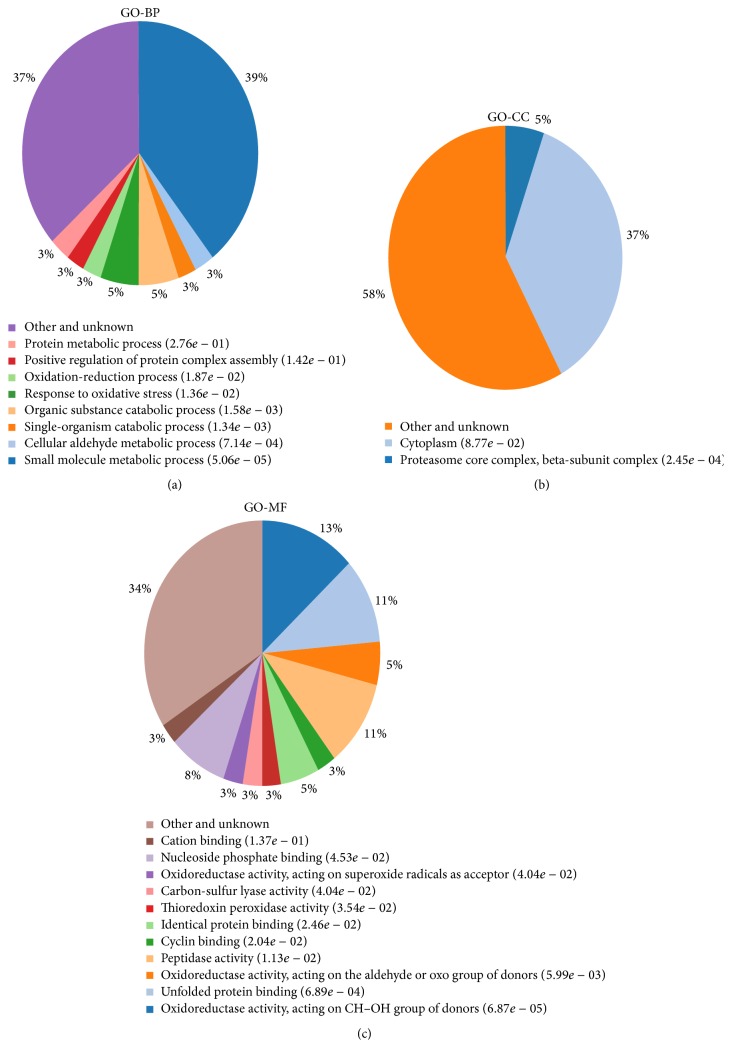
Functional category distribution of the differentially expressed proteins. Pie charts representing the distribution of the 52 identified proteins according to biological function are shown.

In order to understand the roles of proteins associated with mycelial BF formation in this mushroom, the differentially expressed proteins were sorted into different categories based on their functions (Table  S2, Figure 3(a)). As shown in Figure 3, in terms of quantitative changes in BF formation, proteins involved in small molecule metabolic processes, response to oxidative stress, and organic substance catabolic processes accounted for the majority of differentially expressed proteins in 313C. In addition, as is shown in Figure 3(a), the most represented groups of proteins were proteins involved in small molecule metabolic processes (39%), response to oxidative stress (5%), and organic substance catabolic processes (5%), followed by oxidation-reduction processes (3%), single-organism catabolic processes (3%), positive regulation of protein complex assembly (3%), and protein metabolic processes (3%). This finding implies that proteins involved in small molecule metabolic processes may play major roles in 313C under light conditions.

### 3.5. Gene Ontology (GO) Analysis of Differentially Expressed Proteins

To gain further knowledge of the biological functionality of the differentially expressed proteins between 313C and 313W, these proteins were used to perform GO analysis and were assigned to three GO vocabularies: biological process (GO-BP), cellular component (GO-CC), and molecular function (GO-MF). As is shown from the results of GO-BP analysis (Figure 3(a)), DEPs were involved in diverse biological processes. The most highly enriched GO-BP category was small molecule metabolic processes (39%), demonstrating that these processes are of functional importance for brown film formation in* L. edodes*. The second highly enriched GO-BP categories were in response to oxidative stress (5%) and organic substance catabolic processes (5%). As for the GO-MF categories (Figure 3(c)), the top three GO-MFs were oxidoreductase activity acting on CH-OH group of donors (13%), unfolded protein binding (10%), and peptidase activity (10%) (Figure 3(c)). These were followed by nucleoside phosphate binding (8%) and identical protein binding (5%). There were other categories also involved in oxidoreductase activity acting on the aldehyde or oxo group of donors (5%) and oxidoreducatse activity acting on superoxide radicals as acceptors (3%). These molecular functions of oxidoreductase activity had a higher proportion (21%) in BF processes, suggesting that the molecular function category was of functional importance for light-induced BF formation.

### 3.6. Analysis on Selected Transcripts of Differentially Accumulated Proteins

**Figure 4:**
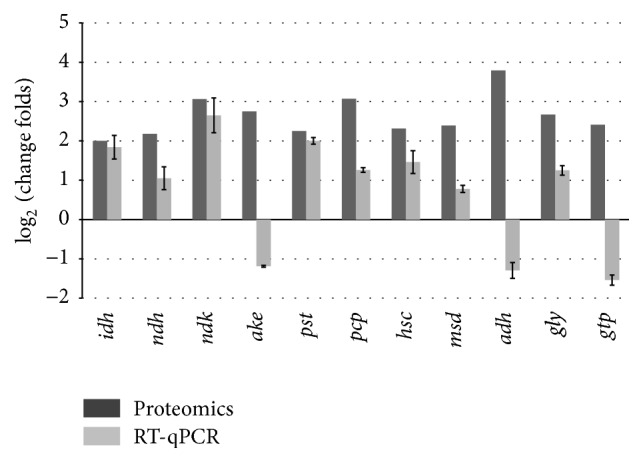
Validation of proteomic data by RT-qPCR. Open and solid bars indicate fold changes obtained from proteomic data and by RT-qPCR, respectively. Error bars represent the mean ± SD of triplicate experiments. The full name of the gene evaluated by qPCR and their fold changes were 3-isopropylmalate dehydrogenase (*idh*, 1.841), NAD-aldehyde dehydrogenase (*ndh*, 1.054), nucleoside diphosphate kinase (*ndk*, 2.645), adenosine kinase (*ake*, −1.189), 20S proteasome subunit (*pst*, 1.998), proteasome component pts1 (*pcp*, 1.26), heat shock cognate 70 (*hsc*, 1.457), manganese superoxide dismutase (*msd*, 1.146), alcohol dehydrogenase (*adh*, −1.293), glyoxalase (*gly*, 1.247), and glutathione S-transferase-like protein (*gtp*, −1.549). The reference gene of qPCR was 18S rRNA.

The proteome results were further confirmed at the transcription level. Eleven proteins, which exhibited upregulated expression, were selected and investigated by RT-qPCR analysis. The genes analyzed included nucleoside diphosphate kinase, proteasome component pts1, manganese superoxide dismutase, and adenosine kinase. The results showed that most proteins (Figure 4) exhibited upregulated expression at the mRNA level comparing 313C to 313W, which correlated to the proteomic data. However, the mRNA level of three genes was different from the mRNA level identified with proteome analysis; the three genes corresponded to adenosine kinase, alcohol dehydrogenase, and glutathione* S*-transferase-like proteins.

## 4. Discussion

As the genome sequences of a variety of organisms are gradually being completed, proteomics will become an increasingly important form of analysis to identify functionally important proteins based on differences in protein expression.

NAD-aldehyde dehydrogenase is an energy metabolism-related enzyme that is differentially expressed during neurosporaxanthin biosynthesis, which is stimulated and regulated by light [15]. The results of our differential proteomic analysis suggest that aldehyde dehydrogenase participates in critical reaction steps in pigment biosynthesis during the process of mycelial BF formation in* L. edodes* and might be regulated by light. In the BF sample (313C), nucleoside diphosphate kinase (NDPK) was upregulated. NDPK regulates various biological processes and signal transduction pathways [16] and can interact with other proteins, including phytochrome [17], catalase [18], and MAP kinase [19]; these proteins function as substrates or regulators in signal transduction pathways. In fungi, light is one of the most important factors for inducing the shape of* Neurospora crassa*, and nucleoside diphosphate kinase-1 (NDK-1), a 15-kDa candidate light signal transduction protein [20, 21], is rapidly phosphorylated after blue light irradiation and may be under the control of WC proteins. In yeast, NDK regulates various signal transduction pathways, such as spore growth and photomorphogenesis [22]. In this analysis, four protein spots corresponding to NDPK were upregulated in 313C, indicating that this enzyme plays an important role in light-induced BF formation. Differential expression of proteins in pathways similar to those of oxidative stress and the light response in plants and other fungi was also observed. Polyketide synthase (PKS) is a large biosynthetic enzyme that contains a variety of active domains that synthesize many complex chemical molecules, some of which have therapeutic and pharmaceutical applications [23], and in some fungi others play important roles in pigment biosynthesis [24–26]. Ketoreductase was significantly upregulated in the BF sample, confirming the involvement of this and related enzymes in complex biological processes and its correlation with light-induced secondary metabolism, which suggests that the pigments in BF likely are comprised of polyketide compounds. We also observed significant upregulation of glutathione S-transferase (GST) in the BF mycelium. GST participates in many reactions involved in the response to biotic and abiotic stresses. Moreover, GSTs can be induced by different types of light in some plants, which is the result of UV light-dependent chalcone synthase signaling pathways [27].

In the present study, differential expression of the 20S proteasome subunit, which plays an essential role in the catalytic activity of the 26S proteasome, was also observed. The proteasome is involved in plant photomorphogenesis [28] and regulates flower development processes [29–31]. Other proteins identified in this study included superoxide dismutase (SOD), a protein whose major role is to transform superoxide anion into hydrogen peroxide and molecular oxygen for defense against cell damage by reactive oxygen species (ROS) [28], and manganese superoxide dismutase (MnSOD), a ubiquitous metalloenzyme also involved in the defense against ROS. Another important DEP was alcohol dehydrogenase (ADH), which plays an important role in regulating alcohol metabolism, anaerobic respiration, and physiological resilience [32]. Anaerobic respiration based on ethanol fermentation is also a major energy supply system under oxidative stress, and ADH activity is significantly increased during hypoxia and in waterlogged environments. Consistent with the results of previous studies, our results suggest that the* L. edodes* mycelium requires more oxygen during BF formation and produces more water, which often leads to submergence and hypoxic stress and, consequently, a need for additional energy supplements, suggesting that the process of BF formation in the mycelium of this mushroom is similar to hypoxia-resistant mechanisms in other crops that involve ethanol fermentation.

In this study, 52 different proteins involved in light-induced BF formation in the* L. edodes* mycelium were identified, providing insights into the mechanism of light-induced BF formation at the proteome level. During light-induced BF formation, a series of physiological and biochemical responses are activated which involve proteins that participate in small molecule metabolic processes and proteins with oxidoreductase activity.

## Supplementary Material

Primers for qRT-PCR to Validate proteomic data in this paper.
