# Supplementary material for: Comparative Proteomic Analysis of Light-Induced Mycelial Brown Film Formation in Lentinula edodes
Source: Biomed Res Int. 2016 Oct 27;2016:5837293. doi: 10.1155/2016/5837293 (PMC5102706; doi:10.1155/2016/5837293)
Supplement: Supplementary file 1 — Primers for qRT-PCR to Validate proteomic data in this paper. [file 5837293.f1.pdf]

Supplementary Materials

Table S1 Primers for qRT-PCR.

Unigene ID	Putative function	Primer
	18S rRNA	F: 5’- AAACGGCTACCACATCCA -3’<br>R: 5’- CACCAGACTTGCCCTCCA-3’
1549	3-isopropylmalate dehydrogenase (*idh*)	F: 5’- AGTGCGGAGCCAGACATTG -3’<br>R: 5’- TCCATCGGTGGGTTCGTC -3’
6339	NAD-aldehyde dehydrogenase (*ndh*)	F: 5’- TCCAAGATGGCTTTCACTCG -3’<br>R:5’- CGGACAGTTTCAAAGCAGTGAG -3’
10501	nucleoside diphosphate kinase (*ndk*)	F: 5’- AGCGTGGACTCGTTGGTGA -3’<br>R:5’- GGTCGGCGTAGTGTTTCTCG -3’
1799	adenosine kinase (*ake*)	F: 5’- TGTGGGATAAAAGGTGCCG -3’<br>R:5’- GCCTGAGATTTCCGCTGTTC -3’
6580	20S proteasome subunit (*pst*)	F: 5’- TGGGTGGAATAGAGGAAGACG -3’<br>R: 5’- CTGTCGTTAGTGGGAGATGTTGA -3’
17492	proteasome component pts1 (*pcp*)	F: 5’- GAACAATGATTTGCGGTTGG -3’<br>R:5’- CTTCCTCGTCAGTCAAGTCCC -3’
10337	heat shock cognate 70 (*hsc*)	F: 5’- GCGATTCCAAGATTGATAAGGC -3’<br>R:5’- CAGCACCGTAGGCAACAGC -3’
13243	manganese superoxide dismutase (*msd*)	F: 5’- CGCACAAGCCTCAACAACC -3’<br>R:5’- CCACTTCCGCCCTTCTCA -3’
16831	alcohol dehydrogenase (*adh*)	F:5’- AAAGTCGGAGACCGTGTTGG -3’<br>R:5’- GCAGGATCGAGTTCTGTGGG -3’
6754	glyoxalase (*gly*)	F:5’- CGAGAATCATCCGTGGGC -3’<br>R:5’- TGGCATCTCAGGACCATACAAG -3’
16026	glutathione S-transferase-like protein (*gtp*)	F:5’- CCAACCATCTCCTTCGTCCA -3’<br>R:5’- AGGTGAAGAGGGCGGGTC -3’

Specific primers of 11 experimentally validated unigene designed for qRT-PCR using Primer Premier 5.0.

Table S2. Differentially expressed protein spots on 2-DE gels from *L. edodes* mycelium samples 313C and 313W as identified by MS/MS.

Spot no.	Unigene No.	Gi no.	Protein name	Protein score	Ion score[a]/Sequence[b]	Fold change(313C/313W)
**Small molecule metabolic process**						
6602	6339	gi|170091726	NAD-aldehyde dehydrogenase	265	31/DIDVAVAAAR; 23/IAFTGSTLTGR; 83/DFDIGDSIQCLR; 81/YYAGWADKITGQTIEVDNK; 90/DAQELAELETLNNGKPVAIAR; 73/IYVQDTVYDKFIDVLVSTVK	0.15
8703	1549	gi|169861624	3-isopropylmalate dehydrogenase	447	56/ELIGGLYFGK;39/IRPEPGLLTLR; 86/VLDLPSAGGYGLR; 81/KVLDLPSAGGYGLR;36/GIANPIGTILSAAMLLR; 122/YSLGLDKPASAIEAAVR; 88IVVLPGDGIGPEVVAEAIK; 108/FDHQLVDSTAMVMVANPR	2.00
7702	8679	gi|299748202	succinate-semialdehyde dehydrogenase	58	58/IYVHSTVYAEFASR	0.12
2804	6339	gi|170091726	NAD-aldehyde dehydrogenase	127	46/IAFTGSTLTGR; 44/DFDIGDSIQCLR; 106/DAQELAELETLNNGKPVAIAR	2.18
6902	10332	gi|299741158	hsp70-like protein	44	26/DIKHWPFEVR; 44/VTHAVVTVPAYFNDAQR	0.14
8605	1203	gi|169852954	methionine adenosyltransferase	104	33/ATIPANLLDER; 32/ELDLQRPNYK; 80/FVIGGPQGDAGLTGR; 9/NNWDLRPGVMVR	0.18
5704	18087	gi|299754101	heat shock protein	645	101/DAGAIAGLDVLR; 86/IQALVSEYFGGR; 65/ARFEEINAAMFK; 50/ARFEEINAAMFK; 111/TTPSYVAFSAEER; 100/AVVTVPAYFNDSQR; 133/IINEPTAAAIAYGLDR; 175/TLSSVTQTTVEVDSLYQGEDFSANITR	0.23
3603	8681	gi|169851323	aspartate kinase homoserine dehydrogenase	293	46/RLPEFDAEFDQMR; 68/TVYFEPTNAITSIEK; 96/FLNESTVGAGLPVISTLK; 89/GYTEPHPADDLNGFDVAR; 72/TILSESNEKPDYESLTAK; 33/EKGYTEPHPADDLNGFDVAR; 56/YPTSHPFATSLGGSDNIIMFHTER	0.08
7801	10131	gi|169848281	diphosphomevalonate	58	14/QIIEIIVR; 17/GLIHTSIGDGPR; 31/SIISIIEELNR;	0.06

			decarboxylase		47/DTTLILPTNSSLSVTLSQDHLR	
2909	1799	gi|169845471	adenosine kinase	526	62/HAPIYEEVVK; 57/EGLQDMYYVK; 144/AHLSSPEISAVLESAK; 143/NYQVTYVAGGASQNAAR; 139/KGEQTGACAVVITGHNR; 165/IVVLTHGAESTVVVTSDAPDAPK; 83/AYYMEGFFLTHGLESALELSQK；	2.75
2910	1799	gi|169845471	adenosine kinase	401	62/HAPIYEEVVK; 50/EGLQDMYYVK; 141/ AHLSSPEISAVLESAK; 148/ NYQVTYVAGGASQNAAR; 105/ KGEQTGACAVVITGHNR; 96/ AYYMEGFFLTHGLESALELSQK	2.14
3704	10337	gi|299753594	heat shock cognate 70	641	64/LVNHFIQEFK; 81/EGKPYIEVEYR; 123/TTPSYVAFTDGER; 85/ANVHEIVLVGGSTR; 65/ARFEELCQDLFR; 76/EGKPYIEVEYRGEK; 140/IDKANVHEIVLVGGSTR; 183/TLSSAAQTSIEIDSLYEGIDFYTSLTR	0.34
3705	10337	gi|299753594	heat shock cognate 70	643	67/LVNHFIQEFK; 83/EGKPYIEVEYR; 123/TTPSYVAFTDGER; 88/ANVHEIVLVGGSTR; 77/ARFEELCQDLFR; 67/EGKPYIEVEYRGEK; 149/IDKANVHEIVLVGGSTR; 156/TLSSAAQTSIEIDSLYEGIDFYTSLTR;	0.19
7404	10337	gi|299753594	heat shock cognate 70	371	34/LTKEEIER; 53/FELSGIPPAPR; 94/NGLESYSYNLR; 109/MVSEAEKYKAEDEAATAR; 129/DNNLLGKFELSGIPPAPR; 161/SETFSTYADNQPGVLIQVFEGER; 125/KSETFSTYADNQPGVLIQVFEGER	2.31
4201	10337	gi|299753594	heat shock cognate 70	334	77/FELSGIPPAPR; 55/YKAEDEAATAR; 81/NGLESYSYNLR; 34/DNNLLGKFELSGIPPAPR; 87/WLDESQEASKEEYEEK; 93/DNNLLGKFELSGIPPAPR; 136/SETFSTYADNQPGVLIQVFEGER	0.35
7402	10337	gi|299753594	heat shock cognate 70	545	81/FELSGIPPAPR; 87/NGLESYSYNLR; 78/DNNLLGKFELSGIPPAPR; 132/DNNLLGKFELSGIPPAPR; 143/GVPQIEVTFDIDANGILNVSAADK； 151/SETFSTYADNQPGVLIQVFEGER; 52/SETFSTYADNQPGVLIQVFEGER； 125/KSETFSTYADNQPGVLIQVFEGER	0.14
3101	439	gi|299752336	glyoxalase I	619	22/VRDPDISVK; 79/FYTEIIGMDLLSK;	0.46

					54/FYTEIIGMDLLSK; 117/TAETASFQFNHTMIR; 44/TAETASFQFNHTMIR; 143/GFGHIAITVDDVEEACAR; 144/HIAFILDPDGYWIEVVPSTLKL; 168/EGVLELTHNHGTETDASFAGYSSGNSEPGK	
2704	1337	gi|310798928	glutamine amidotransferase class-II	175	47/YEPTQQGR; 42/SLYLSLTAGEK; 63/VGPFTPGPGSER; 95/KYFPELPAPTESEDVDNTDVK	2.22
2702	1337	gi|310798928	glutamine amidotransferase class-II	211	42/YEPTQQGR; 64/SLYLSLTAGEK; 61/VGPFTPGPGSER; 123/KYFPELPAPTESEDVDNTDVK;	0.21
9001	16831	gi|189207801	alcohol dehydrogenase	153	88/VIVATAPQGEAIATLLGGLK; 88/KGDLVAVHGLGGLGHLGIQYAK;	3.79
0006	10501	gi|169853921	nucleoside diphosphate kinase	332	72/GKPFFPGLIK; 116/NICHGSDAVESAQK; 39/EQTYIMVKPDGVQR; 91/EQTYIMVKPDGVQR; 23/ VMLGATNPLASAPGTIR; 77/ YMASGPVVAMVWEGLDAVK; 69/YMASGPVVAMVWEGLDAVK	3.06
7102	10501	gi|169853921	nucleoside diphosphate kinase	76	64/GKPFFPGLIK; 38/VMLGATNPLASAPGTIR	2.88
6204	10501	gi|169853921	nucleoside diphosphate kinase	67	64/GKPFFPGLIK; 28/VMLGATNPLASAPGTIR; 54/YMASGPVVAMVWEGLDAVK	2.43
9202	10501	gi|169853921	nucleoside diphosphate kinase	197	53/GDYALAVGR; 66/GKPFFPGLIK; 66/LVQATTEHLEK; 29/VMLGATNPLASAPGTIR; 28/VMLGATNPLASAPGTIR; 88/YMASGPVVAMVWEGLDAVK;	2.10
7204	18648	gi|170085829	phosphopyruvate hydratase	169	43/IALDVASSEFYK; 29/AVANVNDTIAPELIK; 80/IEEETAGSGAFFAGDK; 79/NSGIQIVGDDLTVTNPLR	0.10
3003	6339	gi|170091726	NAD-aldehyde dehydrogenase	57	9/AVHWNIGEK; 53/KEEIFGPVLSIGR; 57/ELGSYALEEYTSVK;	0.05
**Cellular aldehyde metabolic process**						
7703	8970	gi|299740308	aldo-keto reductase	94	31/FGITPQGPR; 57/ELGVTIVAYSPLAR; 53/HIDTAHIYADSEALLGK; 53/LGVETIDLYYQHRPDPK;	0.13
**Single-organism catabolic process**						
9308	5763	gi|82408362	family A1 protease	119	74/ATLDQTTGLLK; 72/FKPNSSVESTR	2.10

**Organic substance catabolic process**						
6504	6580	gi|170089877	20S proteasome subunit	245	17/LIQTMLYSR; 45/QSEGYSIQTR; 108/HIEVGDGLEMFIVLAK; 72/AAGAAQSLVQPFLDNQIYFK; 87/GSPLYGLENIDGVQEMTVTNEGER	2.25
7505	17492	gi|299752963	proteasome component pts1	292	28/FIGNYDISK; 54/FQGGVIVAVDSR; 93/TGPAVFYVDSDGTR; 62/WDLTDEEAQELGR; 97/LHYDGPGETPGAGYGYDIR; 71/LKGDLFSVGSGSTFAYGVLDQGYR;	4.07
**Response to oxidative stress**						
8205	976	gi|299739322	thioredoxin-dependent peroxidase	62	52/LGLDLDLSALAFGTR; 33/KLGLDLDLSALAFGTR	0.41
2202	13243	gi|15042614	manganese superoxide dismutase	463	138/EAVIASFGSLENLQK; 127/VDYLNAIWHVINFDEAQK; 81/VDYLNAIWHVINFDEAQK; 85/VDYLNAIWHVINFDEAQKR; 95/FNGGGHINHSLFWQNLAAPSEK; 179/HHQTYVNALNAAEAAFAQASTTK	2.39
**Oxidation-reduction process**						
8402	6638	gi|170090950	protein disulfide isomerase	173	77/VNFVWIDAIK; 68/WPSFVIQDLEK; 82/QFVSGELKPELK	0.33
Positive regulation of protein complex assembly						
3402	6449	gi|170103737	nucleosome assembly protein	290	111/NHIGLADLITER; 41/YLALQKPLYER; 63/NHIGLADLITERDEGALK; 83/ENIVPSAIPEFWLTALR; 95/LSYLNENEKEYEGKPGFK; 76/SYLYQDEVGYSGDFVYYK	0.22
**Protein metabolic process**						
7203	8798	gi|149689513	small ubiquitin-related modifier	87	24/VGKEPHTIR; 87/VVSSSGEEVFFK; 41/SEEDQQAPPTQEDVK	0.50
**Others and unknown**						
5902	13069	gi|302682544	glycoside hydrolase family 13 protein	147	32/SIYQVMTDR; 14/SIYQVMTDR; 59/LYAYFATLNTFR; 40/QALWLTGYPTGSNK; 69/DVSLLGSFMENHDNAR; 40/DVSLLGSFMENHDNAR	0.07
5601	980	gi|259479840	TPA: ketoreductase (AFU_ortholog ue; AFUA_2G1028 0)	508	85/EALDNGYIVR; 118/ILVTGANGFLGAHIVK; 120/DFLDPAIDGTLNVLR; 103/LSPIPEADPLAGKPFSK; 82/KDFLDPAIDGTLNVLR; 126/AIPPDTVPVFCDVVSTAQIHVK	0.08

8801	11547	gi|169861778	tripeptidyl peptidase A	154	99/AYPDVAAQGEGFQVVVGGR; 75/LFAQPSYQTSAVSAYLAK;	0.49
2203	6372	gi|170086626	linoleate diol synthase	33	33/GLLYPDTFSEER	2.56
9901	6259	gi|299749665	proliferation-associated 2G4	29	29/AGAFPFNIR	0.12
0301	1377	gi|299738240	short-chain dehydrogenase/reductase SDR	561	63/YAAPQYSIR; 46/LGYSIALIAR; 103/GILIFTGATASIR; 91/NNPEWEQNEDVR; 151/LSPEAIASSYVYLASQER; 127/QNIHVAHAIIDGSISTPLSR; 125/IITETNVEGSFAFAHEVLSDFKK	3.61
8002	19905	gi|302682522	carbohydrate-binding module family 13 protein	660	113/SGTILDLSGTDGR; 99/TISGFSDNGGDNQK; 111/WQLEQGQGGHWTFR; 92/WDIYPDDEDSSVFR; 58/WQLEQGQGGHWTFR; 204/NAASGQYLGVGGPFEDGTPLISVDEPVR; 116/TISGFSDNGGDNQKWQLEQGQGGHWTFR; 75/IYVPGAPTALNIDLSDHGNPTPGTIVTLWGK	0.11
3706	6456	gi|299756017	lincomycin-condensing protein lmbA	308	115/HFGSGSLSVADVLAPAIR; 116/GVVSSSQPLATEAGLEILR; 84/LAEEGVPVSEIHSFSWQR; 50/LAEEGVPVSEIHSFSWQR; 64/LAFSDSQYYVTDPDVVNVPVEELLNR	0.10
6503	1450	gi|189190374	NAD dependent epimerase/dehydratase family protein	85	69/SDFETFDIR; 37/ILMTGVTGYIGGTILTR	2.04
2803	980	gi|259479840	TPA: ketoreductase (AFU_orthologue; AFUA_2G10280)	279	57/EALDNGYIVR; 74/GPGTVYQMLQIIAK; 78/DFLDPAIDGTLNVLR; 91/LSPIPEADPLAGKPFSK; 71/KDFLDPAIDGTLNVLR;	2.27
0603	15960	gi|125503211	lipase	208	76/LPDLSHINNK; 112/VGNQAFANLIDSK; 180/EQAQTATAVLAAVQQAMSAH; 121/EDPVPILPGEFLGFVHPSGEK; 85/LPDLSHINNKEDPVPILPGEFLGFVHPSGEK	2.06
8802	11547	gi|169861778	tripeptidyl peptidase A	356	159/AYPDVAAQGEGFQVVVGGR; 91/LFAQPSYQTSAVSAYLAK; 154/IGITGYLEQFASNSDLATFVK	0.16
6502	16026	gi|75911207	glutathione	42	23/FFIDTFANK; 39/FGPALFTFQSGK	2.41

			S-transferase-like protein			
9101	17492	gi|299752963	proteasome component pts1	30	9/TGPAVFYVDSDGTR; 30/LHYDGPGETPGAGYGYDIR	3.07
5404	6754	gi|310791466	glyoxalase	229	40/RLPLPFFCGDVTPR; 113/SLDYIIGYSGVTSADAER; 87/APGWIDYAFLGNGSETVR; 54/VPNEPPAIFEHPCTALGIAYVR	2.67
3302	15973	gi|160184939	competence/damage-inducible protein CinA	43	43/GPDEQIVIGLASHLR	0.46
2904	16388	gi|260907550	FG-GAP repeat-containing protein	28	28/IQVAGFPIIVR	0.33

[a]The score of matched peptides; [b]The sequence of matched peptides
